# HIV Care Continuum Services for People Who Inject Drugs in Kazakhstan During COVID-19: A Qualitative Study of Service Provider Perspectives

**DOI:** 10.9745/GHSP-D-21-00619

**Published:** 2022-04-28

**Authors:** Tara McCrimmon, Anne Sundelson, Meruyert Darisheva, Louisa Gilbert, Timothy Hunt, Assel Terlikbayeva, Sholpan Primbetova, Nabila El-Bassel

**Affiliations:** a Columbia University Mailman School of Public Health, New York, NY, USA.; b Global Health Research Center of Central Asia, Almaty, Kazakhstan.; c Columbia University School of Social Work, New York, NY, USA.

## Abstract

**Introduction:**

The coronavirus disease (COVID-19) pandemic and resulting lockdowns have disrupted health care service delivery globally. This includes disruptions in harm reduction and HIV service delivery for people who inject drugs (PWID), a population at high risk for not only COVID-19 but also poor HIV and drug-treatment access. However, little is known about these issues in Kazakhstan. We examined harm reduction provider experiences with delivering services and regulatory changes during the COVID-19 pandemic.

**Methods:**

We conducted in-depth interviews with 24 nurses, social workers, and doctors serving both HIV-positive and HIV-negative PWID at 13 needle and syringe programs (NSPs) and 4 AIDS Centers (HIV treatments centers) in Kazakhstan from May to August 2020. Participants were asked how the COVID-19 pandemic had impacted their PWID clients' risks, their organizational environment, and the services offered to PWID over the prior 3–6 months. Thematic content analysis was used to elicit findings.

**Findings:**

The COVID-19 pandemic considerably impacted NSP and AIDS Center operations. Participants perceived high risks of severe acute respiratory syndrome coronavirus-2 (SARS-CoV-2) infection for themselves and their clients, as well as pandemic-related increases in substance use and HIV risks for clients. Organizations instituted several policy and regulatory changes to adapt to the pandemic, most notably tasking NSPs with delivering HIV medications; these changes necessitated new roles and responsibilities for many providers. Despite this stressful changing environment and increased service demands, participants still shared examples of persistence and resilience as they worked to meet client needs during these challenging times.

**Discussion:**

NSPs in Kazakhstan are well-positioned to reach key populations with crucial information and flexible services during the COVID-19 pandemic. However, they need recognition as essential organizations and additional equipment and staff support to protect staff and clients, maintain pandemic-related regulatory changes, and address additional challenges such as overdose prevention among clients.

## Abstract (precis)

Needle and syringe programs (NSPs) in Kazakhstan have been crucial in providing care for people who inject drugs (PWID) during the COVID-19 pandemic. Additional supports and investments are needed to ensure that NSPs can continue to reach these marginalized populations while traditional medical systems are under strain.

## Teaser

**Key Findings:**

- As organizations implemented policy and regulatory changes in response to the pandemic, service providers at needle and syringe programs (NSPs) and AIDS Centers (HIV treatments centers) demonstrated flexibility and persistence in taking on new roles and responsibilities while continuing to meet the needs of people who inject drugs (PWID).
- NSPs are well-positioned to provide services to marginalized groups during the pandemic because they have community connections and flexibility that traditional health facilities may not have.
- Providers at NSPs were hampered by a lack of resources, limited personal protective equipment access, low salaries, and the designation of NSPs as nonessential services, which forced NSPs to close during the peaks of the pandemic. This underinvestment in NSPs undermines their potential to provide care to key populations during the pandemic.

**Key Implications:**

- Differentiation-of-care approaches are essential to continue reaching marginalized groups during a crisis on the scale of the pandemic, which deeply impacted service provision through traditional medical systems.
- Kazakhstan's national health care policy makers should increase resources and support for NSP operations during COVID-19 to ensure that PWID continue to receive necessary services for HIV and harm reduction.

## BACKGROUND

In the 6 months between Kazakhstan's first identified case of coronavirus disease (COVID-19) (which was registered on March 13, 2020) and the end of August 2020, the country experienced 105,795 infections and 1,523 deaths.^1^ During this time, regional governments instituted a series of restrictions and other measures (see examples from 3 regions in the Figure) to prevent disease spread, leading to profound disruptions in standard medical and social service delivery to all Kazakhstani citizens, particularly those in marginalized populations. Kazakhstan has an estimated 94,600 people who inject drugs (PWID),^2^ a population of particular concern in this pandemic.

**FIGURE:**
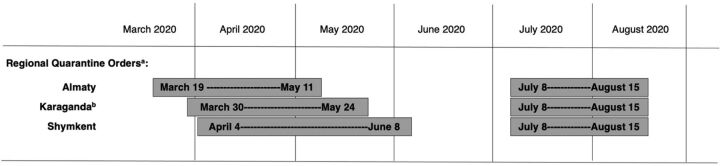
COVID-19-Related Regulations in 3 Regions of Kazakhstan, March 2020–August 2020 Abbreviation: COVID, coronavirus disease. ^a^ Includes: Nonessential services closed, public transportation limited to essential workers, restrictions on public gatherings and movements. ^b^ Includes: study cities of Karaganda and Temirtau.

Although there are no data about the impact of COVID-19 among PWID in Kazakhstan, global research suggests that PWID are at increased risk of COVID-19 illness and poor outcomes due to medical comorbidities, social and structural factors, and substance use related-stigma that may deprioritize them for COVID-related care.^3^^–^^5^ While the full impacts of COVID-19 on PWID will take time to ascertain, preliminary data suggest these predictions are accurate. A study among people who use drugs in the United States found that those with recent substance use (in the past year) had a higher risk of infection with severe acute respiratory syndrome coronavirus-2 (SARS-CoV-2) compared to those without.^6^

Moreover, global data suggest that the pandemic and associated lockdowns may increase PWID's vulnerability to HIV-related and drug use-related harms. Social isolation during lockdowns has been linked to increased risk of substance use,^7^ as have the economic and mental health tolls of the pandemic.^8^^,^^9^ Studies have shown that people with substance use disorders who have HIV were more likely to have used illicit substances during the pandemic than before; they also reported reduced confidence in their ability to stay sober.^10^ Disruptions in supply chains can lead to drug substitutions or encourage injection of substances that were previously consumed in another modality, increasing the risk of overdose.^3^^,^^9^ Substance use in isolation may also result in a higher risk of fatal overdose.^3^^,^^11^ Indeed, provisional data from the U.S. Centers for Disease Control and Prevention indicates that U.S. overdose deaths increased more than 30% between 2019 and 2020.^12^ Finally, stay-at-home orders and other lockdown measures may make it challenging for PWID to seek medical care or harm reduction services, including HIV prevention services. A survey of PWID in the United Kingdom revealed difficulties in accessing HIV testing, hepatitis testing, and clean syringes for injection during the pandemic.^13^ These data show the importance of maintaining continuity of care for PWID during the COVID-19 pandemic and the need for additional research regarding the challenges that organizations may face in doing so.

Even before the pandemic, community-based harm reduction programs such as needle and syringe programs (NSPs) were recognized as promising venues for a differentiation-of-care approach. This approach provides community-based and accessible alternatives to traditional medical systems such as hospitals for key populations at risk of HIV.^14^^,^^15^ Kazakhstan's 130 NSPs are the main source of harm reduction service access for PWID. These programs, available through primary health care settings (polyclinics), nongovernmental organizations (NGOs), and AIDS Centers (HIV treatment centers), are responsible for anonymous (without official identification) distribution of injection materials, condoms and lubricants, rapid HIV testing, and referrals to HIV treatment and other services. While Kazakhstan's traditional medical system is overwhelmed with the COVID-19 response, these organizations may play a crucial role in providing continuity of services for PWID. However, harm reduction programs globally are facing several pandemic-related challenges, including staff redeployment to COVID-19 services,^16^ maintaining client social distancing,^17^ or in some cases, the complete or partial suspension of services due to shutdowns, funding restrictions, and staffing gaps.^16^ Additionally, harm reduction service providers may suffer from burnout and compassion fatigue, leading to reduced quality of care and workforce dropouts.^18^^–^^20^

Kazakhstan's 130 needle and syringe programs are the main source of harm reduction service access for people who inject drugs.

Despite these pressures, there are several examples of harm reduction programs adapting their services to continue existing operations and better serve their clients during the pandemic. In Australia, 2 supervised injection facilities implemented on-site COVID-19 testing among clients.^21^ In Los Angeles, a harm reduction service implemented a telephone booth system for on-site yet socially distanced consultations and coordinated pharmacy-based dispensation of medication for opioid use disorder (MOUD).^22^ In Rhode Island, regulatory changes at a federal level allowed a harm reduction organization to coordinate telephone-based buprenorphine prescription.^23^ In Scotland, a shelter provided take-home naloxone to its clients.^17^ Telehealth services in the United States and Russia demonstrated success in retaining those with substance use disorder in buprenorphine treatment and other services.^24^^,^^25^

Although these examples show the promise of innovative solutions in maintaining continuity of care for PWID, the majority come from a high-income country context. Despite high rates of injection drug use in Eastern Europe and Central Asia, little research has examined the impact of COVID-19 on service delivery for PWID in this region. We address this gap through a qualitative exploration of COVID-19-related changes in services in NSPs and HIV treatment centers in Kazakhstan. We sought to understand service providers' perceptions regarding COVID-19 information and risks of SARS-CoV-2 infection, the impact of the pandemic on HIV and drug use risks, regulatory and service changes in harm reduction and HIV care settings, and the impact of these changes on providers.

Little research has examined the impact of COVID-19 on service delivery for PWID in Eastern Europe and Central Asia.

## METHODS

We conducted in-depth interviews with service providers in a larger HIV implementation research study, Bridge, which took place between January 2017 and April 2020.^26^ This parent study was designed to evaluate the effectiveness and implementation of the Bridge intervention in 24 NSPs. Bridge was based on a differentiation-of-care approach to HIV care for PWID by enhancing the organizational and staff capacities of NSPs to engage PWID in regular HIV testing, identify HIV-positive PWID, and enhance linkage to HIV care and treatment. Bridge was implemented in 4 Kazakhstani cities: Almaty, Shymkent, Karaganda, and Temirtau. These cities were selected for their large populations of PWID, sufficient numbers of NSPs, and estimated numbers of HIV-positive PWID who remained unlinked to HIV care. Service providers from 24 NSPs and 4 AIDS Centers participated in the parent study. We conducted in-depth interviews to elicit participant reflections on the full study. We stratified participants by their professional role (nurse, doctor, social worker, or leadership position). Of the 170 providers in the full study, we randomly selected 24 and invited them to participate in the in-depth interviews. Organizational and participant characteristics are summarized in the Table.

**TABLE.** Characteristics of In-Depth Interview Organizations and Participants Included in Qualitative Study of Service Provider Perspectives, Kazakhstan

	Almaty	Shymkent	Karaganda/Temirtau	Total
AIDS center	1	1	2	4
Total NSPs	4	4	5	13
AIDS center-based	0	1	3	4
Polyclinic-based	4	1	2	7
NGO-based	0	2	0	2
Total Organizations	5	4	4	13
Participant characteristics				
Place of Employment				
AIDS center	3	3	4	10
Total NSPs	4	5	5	14
AIDS center-based	0	1	3	4
Polyclinic-based	4	1	2	7
NGO-based	0	3	0	3
Role				
Doctor	3	3	4	10
Nurse	4	2	5	11
Social worker	0	2	0	2
NGO director	0	1	0	1
Gender				
Men	2	1	1	4
Women	5	7	8	20
Total Participants	7	8	9	24

Abbreviations: NSP, needle and syringe program; NGO, nongovernmental organization.

We developed semistructured interview guides for the parent study following the Consolidated Framework for Implementation Research, the guiding framework for the larger study.^26^ We then added questions regarding the organizational burden of COVID-19 in the past 3–6 months; participants' responses to these questions provided the data which are the focus of the present analysis. Between May 2020 and August 2020, 3 trained research assistants conducted all interviews using the web-based videoconferencing platform Zoom. Interviews were conducted in Russian, transcribed from audio recordings, then translated into English.

Two researchers were responsible for codebook refinement and analysis based on English-language transcripts. Both researchers were native English speakers with proficiency in Russian; native Russian-speaking bilingual members of the research team provided additional translation and contextual clarifications as they arose. A set of codes related to COVID-19 were inductively identified using a sample of 7 transcripts. These codes were discussed between the 2 researchers and reviewed by the broader study team. Revision descriptions and justifications were recorded in the codebook log. Researchers used iterative updated versions of the codebook in each subsequent coding. Once a finalized COVID-19 coding framework was established and approved by the full research team, the same researchers conducted coding using NVivo 12. Coded data were analyzed using a thematic analysis approach to elicit patterns in how organizations responded to the COVID-19 pandemic and related restrictions, including shifts in staffing and organizational guidelines, with a focus on increased staff burden. The 2 researchers summarized themes across interviews, then met with the full research team to share, discuss, and further develop findings.

### Ethics Approval

All study protocols received approval from the Institutional Review Boards at Columbia University and by the ethics committee of the Kazakhstan School of Public Health (KSPH). All selected providers agreed to participate in the in-depth interviews and gave verbal consent to participate.

## FINDINGS

The COVID-19 pandemic considerably impacted NSP and AIDS Center operations. Here, we include a representative sample of participant responses to describe the findings related to (1) COVID-19 information, perceived risks, and safety measures; (2) pandemic-related increases in drug use and overdose/HIV risks for PWID and people living with HIV (PLHIV); (3) changes in harm reduction and HIV service delivery; and (4) the toll that these had on service providers.

### COVID-19 Information and Risk Perceptions

Participants received general information about COVID-19 from a variety of sources, both official and unofficial, including the Ministry of Health, AIDS Center management, colleagues, internet, television, and social media. While some participants felt that they had sufficient information, several discussed obstacles to staying adequately informed, including a lack of information, too much information, a rapidly changing epidemiological situation, isolation from colleagues/sources of information, and concerns about the trustworthiness of information.

*I think that no one has the most adequate information… no one can definitively say what the situation is now… I watch [TV channel], also, Internet, WhatsApp groups. But the TV is no longer truthful. That's the way [it is].* —Nurse, NSP, Almaty

*Now, since we have a lot of sources of information, it's just some type of informational intoxication. You try, of course, to choose information only from trusted sources.* —Doctor, AIDS Center, Temirtau

Participants often mentioned that misinformation about COVID-19 was a widespread and concerning issue, and some indicated that their NSP clients believed rumors about COVID-19. The main content of the misinformation appeared to be the idea that COVID-19 was not real and/or was a manufactured bioweapon.

*Unfortunately, there is no such information on how COVID-19 affects PWID and PLHIV*. —Doctor, AIDS center, Shymkent

This response represented the views of many providers, who noted a lack of official information regarding COVID-19 risks among these vulnerable client groups. Despite this, several expressed their beliefs that their PWID clients, particularly those living with HIV, were at increased risk for SARS-CoV-2 infection. Many stated that their HIV-positive clients had increased biological susceptibility to the virus due to their immunocompromised state.

Many providers noted a lack of official information regarding COVID-19 risks among these vulnerable client groups.

Others noted that PWID have comorbidities that may worsen disease outcomes.

*All PWID who use drugs have weak immunity. Moreover, everyone has concomitant diseases. Therefore, they are at risk.* —Nurse, AIDS Center-based NSP, Shymkent

*You understand that HIV infection is associated with immunity, naturally, such people are more at risk of getting coronavirus, therefore, of course, the important point is that people living with HIV receive [ART] without interruption.* —Doctor, AIDS Center, Temirtau

Participants expressed mixed views on their clients' protective measures from COVID-19. Some described great cautions taken by clients to avoid infection. However, many others were critical of clients for not observing distancing or mask wearing. Some believed PWID lacked self-control when under the influence of drugs, increasing their risk of SARS-CoV-2 infection.

*When they inject, they do not control their actions.* —Nurse, NSP, Karaganda

*The clients don't always come in wearing masks. Many of our clients just don't care, and they don't think about the personal protective equipment.* —Director, NGO, Shymkent

Others noted that economic insecurity put their PWID clients at higher risk for both SARS-CoV-2 infection and negative COVID-19 outcomes, as they were unable to purchase masks, register for health care without state-issued identification documents (which many PWID struggle to maintain amidst socioeconomic instability), or afford medications.

*Naturally, they are more vulnerable. They are unable to buy masks, they cannot buy sanitizers. If they get sick, many of them are not able to buy basic medicines… Without [identification] documents, as you know, they are not attached to the clinic, to the medical institutions. And as a result, they may not even receive basic medical care.* —Nurse, NSP, Temirtau

Participants described a range of protective measures for staff and visitors, including staff rotations, limiting clients inside and in waiting areas, contact tracing, temperature checks, and disinfecting (air filters, disinfectant wipes) in between clients. Some participants received enough personal protective equipment (PPE) from their employers to distribute masks among clients; others reported having to purchase PPE for themselves.

*We all worked in turn, one person at a time, … because the situation worsened; orders were received from management to separate the team as much as possible.* —Doctor, AIDS Center, Temirtau

*But the pandemic also affects the schedule… We clean everywhere, all day, and wipe our hands constantly. We are very serious about it. This affects the schedule; more attention is separated for this.* —Nurse, NSP, Karaganda

Participants acknowledged that these protective measures added to their workload, changed the atmosphere of the NSPs, and discouraged PWID client visits. One participant noted that PWID clients were uncomfortable with providing their names for COVID-19-related contact tracing when they visited the polyclinic where the NSP is located. Others noted that PWID, who usually visit in groups, were less comfortable coming individually.

*They register their last names, first names… they write everything, and our PWID do not want to be advertised… Before…when there was no epidemic, I could let them through the back door and provide services and then let them go, but now I cannot do this.* —Nurse, NSP, Karaganda

*They come in separately… they used to go there [together]… they liked to communicate, but now [if] a person is coming, they ask him to get syringes, condoms*. —Nurse, NSP, Karaganda

In addition to implementing these new protective measures in the workplace, NSP staff also described providing COVID-19 information to clients. Participants disseminated information through outreach workers, hung informational posters, and made themselves available to PWID with questions by phone and in person. One participant mentioned that it was difficult to keep PWID informed who didn't have access to smartphones and that their NSP was undertaking efforts to overcome this barrier through printed media.

*They even call [about COVID-19] and ask: “Is it dangerous? How? What about it?" It feels like they have me, well, like, the main source of information.* —Nurse, NSP, Karaganda

*We are trying to get that information to our clients, but unfortunately, not everyone has a smartphone. They all use simple phones and so it is very difficult to provide the information in an electronic format. But we are trying to print them out in our office to hang up. Our whole office has COVID-19 informational posters hung up.* —Director, NGO, Shymkent

### Client Overdose and HIV Risks

Participants recognized that COVID-19 itself was only one risk facing their clients. Local and national response measures to the pandemic impacted their clients economically, as well as in terms of drug use and HIV. A few reported clients resorting to stealing, selling property, and extortion to obtain money to live or buy drugs.

Participants expressed diverse views regarding the impact of the pandemic on injection drug use and overdose risk. Many said they were unaware of this issue or emphasized that their primary role and responsibility was related to HIV risk reduction.

*The main thing is to stop the spread of HIV infection and hepatitis B, C… I don't talk to PWID about overdoses.* —Doctor, AIDS Center, Shymkent

While some respondents believed that access to drugs was more difficult in the pandemic, others maintained that their clients were finding ways to access drugs regardless. Participants stated that supply chain issues encouraged PWID to switch drugs (particularly from opioids to stimulants), mix drugs, or use alcohol. Several recognized the increased risk of overdose that came with polysubstance use or use of unfamiliar drugs.

*[Overdose] is definitely increasing. Their bodies are already adjusted to the opium, but then they start to mix new drugs and then overdoses start occurring*. —Social worker, NSP, Shymkent

Others noted additional factors contributing to overdose risk or death from overdose, including increased substance use due to the mental health impacts of isolation, use in isolation without responders available in case of overdose, and resources like ambulances being occupied with COVID-19 and unable to respond to overdoses.

*You won't go anywhere; you won't talk to anyone. You are at home all the time within 4 walls. Maybe someone is oppressed by such an atmosphere, and a person could start using more often … it could lead to more frequent overdoses.* —Doctor, AIDS Center, Temirtau

*Because the ambulance is actually very busy right now… even if he got through, he must wait … it sometimes takes several hours.* —Doctor, AIDS Center, Temirtau

Providers noted additional factors contributing to overdose risk or death from overdose, including increased substance use due to the mental health impacts of isolation and use in isolation without responders available in case of overdose.

Some participants in Almaty mentioned that their clients had previously accessed naloxone for overdose prevention through a United Nations Office of Drugs and Crime-World Health Organization project that had ended the year prior;^27^ however, none described ongoing efforts to distribute naloxone among PWID. Naloxone distribution is insufficient and underprioritized in Kazakhstan, limited to first responders and donor-funded projects such as this.

Finally, a few participants expressed the belief that the pandemic provided an opportunity for PWID to reflect upon their drug use behaviors and possibly change them.

*This period of self-isolation, all this situation, let us rethink life… I don't know how they changed their behavior or not, but many were given time to change themselves.* —Doctor, AIDS Center, Temirtau

Participants also noted clients' increased risks of HIV acquisition during this period, particularly if they quarantined together.

*If they gathered in one room all together, then this year there may be many [HIV] positive people… after all, they are injecting each other.* —Nurse, NSP, Almaty

One participant described how public transit restrictions (Figure) and resulting police control of movement through the city could make PWID reluctant to seek risk reduction services.

*[Police] could stop any citizen, not just PWID, and explain the limitations. Because the movement limitations meant you could only go to the pharmacy and to buy groceries. So, of course, with PWID, this affects whether they receive clean syringes.* —Doctor, AIDS Center, Almaty

### Changes in Harm Reduction and HIV Service Delivery and Regulations

NSP staff described a substantial shift in how harm reduction services were delivered in response to national and regional lockdowns, directives from parent organizations (polyclinics, AIDS Centers, and NGOs) as well as local transportation limitations and movement restriction zones. At the most extreme, services were suspended entirely; other times, staff were directed to work remotely. In addition, there were individual cases when an organization or a portion of its staff would quarantine due to SARS-CoV-2 exposure. Many NSP staff viewed this as a period of stagnation.

NSP staff described a substantial shift in how harm reduction services were delivered in response to national and regional lockdowns.

Participants noted a few cases of the pandemic resulting in the delay of planned services and opportunities. Notably, in Shymkent, a participant described how plans for methadone maintenance treatment (the only form of MOUD approved for use in Kazakhstan) had been postponed as everyone turned their attention to the pandemic.

*[Methadone maintenance treatment] is … postponed in connection to the pandemic. We haven't had therapy since then, because of quarantine…. the [SARS-CoV-2] infection flare-up increased, so we are concentrating on that. These programs are on freeze.* —Director, NGO, Shymkent

Many participants mentioned that workers at NSPs (including outreach workers and nurses) were asked to take leave from work due to COVID-19-related closures and quarantines.

*Many large [medical] centers were quarantined, so everything was suspended, and the office room was closed. Some were sent on non-paid leave, others were sent on vacation, to be honest, I can't tell you exactly whether people came to the clinic.* —Nurse, NSP, Almaty

Participants described the impossibility of remote work for nurses and outreach workers. Several described arranging individual meetings with clients despite officially working remotely.

*If I work in the “remote office” how will they exchange syringes? They will have panic. When I go on vacation, I come 2 or 3 times a week, hand out syringes to them. They cannot be left without attention.* —Nurse, NSP, Karaganda

Participants employed by the AIDS Center reported fewer disruptions in HIV care services in their facilities. Many attributed this to the fact that their organizations, unlike NSPs, were considered “medical” facilities and therefore were permitted to continue operations as essential services.

NSPs and AIDS Centers implemented new policies and procedures to facilitate operations during the pandemic. Many of these entailed additional or shifting responsibilities for staff. Participants described several instances of staff reallocations. In Temirtau, AIDS Center doctors were reallocated to COVID-19-dedicated hospitals, and nurses took over their responsibilities in the AIDS Center. In both Karaganda and Shymkent, outreach work at polyclinic-based NSPs were shifted to NGOs to allow polyclinics to focus their energies on COVID-19 cases.

*We no longer provide outreach services… [NGO] provides them with syringes and condoms. We only test for HIV, a rapid test and take blood samples from them.* —Nurse, NSP, Karaganda

By some participants' accounts, the COVID-19 pandemic provided the opportunity to overcome preexisting regulations related to secondary exchange of materials and longer-term provision and home delivery for antiretroviral therapy (ART).

*Previously, we gave individual syringes and condoms to [PWID] who came, and now we agree that they pass them on to each other.* —Nurse, NSP, Karaganda

*We might give [ART] for a long period of time … so that people aren't risking their health on public transportation to get all the way to the AIDS Center.* —Doctor, AIDS Center, Almaty

However, the most notable policy regulation that was overcome occurred in Karaganda, where polyclinic-based NSPs were charged with distributing ART (previously only available at the dispensary department at the AIDS Center itself). Two NSP nurses described their experience with providing medications to their PWID-PLHIV clients.

*[The AIDS Center] brought medicines to NSPs for those who live far away. We still have transportation there… It's easier for [clients] to get there.* —Nurse, NSP, Karaganda

*I distributed a lot of medicines to my patients, that is, we had a connection with our treatment department. They said that it was necessary to give this to patients who were running out of medicine*. —Nurse, NSP, Karaganda

When asked whether this service could be provided beyond the end of the pandemic, the 2 nurses expressed doubts.

*I don't know. I cannot answer that. We could, of course, distribute medicines, but they probably had better come there to the AIDS Center, talk to a doctor, tell them what side effects they have, if they can increase or decrease the dose. We can transfer, of course, distribute and transfer these ART medicines, right? It will probably be more convenient for them to come there, take a [viral load test], and talk to a doctor. It will probably be more effective for them.* —Nurse, NSP, Karaganda

*Of course, it can, because the NSP is in [neighborhood], it is more convenient… But it won't be possible all the time… [the doctor] must find out about his condition.* —Nurse, NSP, Karaganda

NSP nurses in Karaganda and doctors (elsewhere) described increased responsibility for home delivery of ART for clients, given that their medical licenses permitted them to move around the city and through travel checkpoints.

*Since I had a certificate that I could move, I went to distant places myself, brought them medicine. I had a shortened working day and then I took all this and called up that I would come and go deliver medicines.* —Nurse, NSP, Karaganda

*We had clients who, during the restrictive measures, remained outside the checkpoints. The delivery of [ART] to checkpoints was organized for such patients, where they received drugs and signed for their receipt. At the time of the ban on movement in the city, patients who ran out of [ART], they contacted their doctors. They left the coordinates with their location; the doctors organized the delivery of ART for them.* —Doctor, AIDS Center, Shymkent

Providers had mixed experiences and opinions on telemedicine, which in these interviews was defined as the remote delivery of services by either phone or internet. Particularly among AIDS Center providers, telemedicine was noted as an opportunity for interprofessional communication and learning, rather than direct communication with clients. Participants expressed the view that telemedicine was particularly difficult to use with PWID, given both their lack of access and familiarity with technology.

*Not all clients have … smartphones with video cameras. There are some clients, especially problem clients, who have low adherence, they generally do not have access to telemedicine, purchase specialized mobile devices, and pay for Internet services.* —Doctor, AIDS Center, Shymkent

Providers expressed that telemedicine was particularly difficult to use with PWID, given both their lack of access and familiarity with technology.

Many noted that telemedicine was a useful tool but cannot cover all service provision. Some noted that it was effective for continuing to monitor clients but not for making changes to their medication regime, or anything that required a visual diagnosis.

*It's kind of just consultations for our clients, over the phone, online… Kind of like a hotline… It's possible that the client doesn't receive information on a service that they would really like to receive.* —Doctor, AIDS Center, Almaty

### Impacts on Service Providers

Participants frequently discussed their own risk of developing COVID-19 as well as actual cases of illness among providers. Several highlighted factors that increased their risk of SARS-CoV-2 infection, including a lack of PPE.

*More than half of the clinic [staff] got infected. Many are on sick leave, and they come to the office. Even many are not allowed, thermal imagers do not work… This is also scary. Now everybody lives in fear.* —Nurse, NSP, Karaganda

*Our partners have been under a lot of stress the past 3–4 months, because of the deficit of personal protective equipment. Not because there weren't any, but because our partners sometimes needed to buy it themselves… The situation in Shymkent right now is stressful. Unfortunately, a flare-up of infections increased.* —Director, NGO, Shymkent

Furthermore, the previously described changes in policies and procedures represented a substantial shift from the regular responsibilities of harm reduction and HIV service provision. Several participants explained that, in conjunction with the pandemic overall, these new responsibilities entailed providers working long/nontraditional hours, under increased stress and at high risk.

*At all times, the nurses and doctor are on call with our patients and outreach workers. They are working in the evening, and even at night. …[Staff] take the situation seriously and feel the weight of it everywhere. Of course, this psychological and physical weight increased.* —Nurse, NSP, Karaganda

Participants also expressed financial concerns related to role reassignment and lack of official support for those who may become ill with COVID-19 through their continued work during this time.

*They have very good bonuses during the COVID-19 pandemic… I now work under an agreement with the provisional center. And after I finish this work, I will return to my hospital, where I'll do the same work, and even more. And the salary will be as small as it was; it worries me very much.* —Nurse, NSP, Shymkent

*I got sick with pneumonia… We were treated on our own wallet, not at any hospital, but we went and bought medicine on our own. So, our partners aren't the only people at risk, but their families are too. The low pay cannot always cover the needs of our partners' families. We are working in these stressful conditions.* —Director, NGO, Shymkent

Despite the fear of infection, actual cases of COVID-19, and increased responsibilities, participants exhibited resilience, adaptability, stoicism, and dedication to continuing to serve clients.

*Maybe, sometimes [nurses] were busy doing other tasks. But whenever a client came in, they would make time for them. So, these medical workers were still dedicated to preventative care, and they were in the medical organizations from morning to night.* —Doctor, AIDS Center, Almaty

*Our nurses, they showed their knowledge, they really showed it… They all showed their qualifications. They were able to work without doctors. They consulted when they had difficulties, but… they knew that we were very involved in difficult situations…* —Doctor, AIDS Center, Temirtau

Finally, nurses at NSPs described how outreach workers served as a key bridge between their organization and their clients during the pandemic.

*In clinics, we only accept people with an urgent problem. Our clients are brought in by outreach workers. They call us in advance. I meet them at the clinic…* —Nurse, NSP, Almaty

*More people started to connect with the outreach workers over the phone. And of course, the outreach workers are on the phone more, so that they can negotiate to allow the patients to come in.* —Nurse, NSP, Karaganda

## DISCUSSION

This study represents a novel investigation of COVID-19-related impacts on harm reduction and HIV services in the Central Asian region. Service providers described the substantial impact of the pandemic on service delivery for PWID at NSPs and AIDS Centers. Findings show that providers operated within a stressful and changing environment, with threats to their own health as well as the health and well-being of their clients. Yet despite these pressures, most providers adapted to shifting regulations, and readily assumed new roles and responsibilities. Their accounts underscore their flexibility and resilience in continuing to serve PWID.

As in other parts of the world,^17^^,^^21^^–^^25^ the harm reduction organizations in Kazakhstan employed a range of innovative solutions to maintain continuity of care for PWID. These included safety precautions and flexible distribution of materials and medication. Changes in regulations, such as those that allowed for NSPs to distribute ART, were employed to ease client access to care. And as in other regions, we saw evidence of provider stress and burnout.^18^^–^^20^

Harm reduction organizations in Kazakhstan employed a range of innovative solutions to maintain continuity of care for PWID.

Our findings highlight some challenges unique to Kazakhstan. Despite perceiving an increased overdose risk for clients during lockdowns, providers described delays in MOUD service expansion and hesitancy to discuss overdose with clients. Take-home naloxone distribution among PWID, which was shown to have success in Almaty even before the pandemic^27^ was not scaled up to meet this increased need. A lack of surveillance data on fatal and nonfatal overdose deaths in Kazakhstan impedes the critical need for data-driven overdose prevention efforts and makes it difficult to ascertain whether the incidence of fatal and non-fatal drug overdoses has increased during the COVID-19 pandemic. While harm reduction programs in other countries have adopted new technologies for telemedicine, providers saw limited potential for this in Kazakhstan, given that their clients could not access the technology that might make this an effective mode of service delivery.

A lack of resources was another recurring theme; providers described limited access to PPE, low salaries, and the designation of NSPs as nonmedical (nonessential) services, which entailed forced closures during the peaks of the pandemic. This underinvestment into NSPs is particularly concerning as it undermines their potential to provide differentiated care to key populations. Participants' descriptions of COVID-19-related educational efforts demonstrate that NSPs are well-positioned to reach marginalized groups with vital information and services in a time of crisis. The service and role adaptations described also suggest that NSPs have the flexibility that traditional medical systems may not; and yet as many participants reported, NSPs were at times deemed non-medical, therefore non-essential and temporarily closed, or their workers were sent on unpaid leave.

The underinvestment into NSPs and lack of resources undermines their potential to provide differentiated care to key populations.

Finally, our findings raise concerns about the role of provider stigma faced by PWID clients in these settings. Participants were frustrated by their clients' carelessness regarding their health and expressed hope that self-reflection during the pandemic could change their clients' drug use behaviors. These findings are consistent with prior ones from the same settings in Kazakhstan that showed high endorsement of stigmatizing attitudes toward PWID living with HIV, including beliefs that clients' irresponsible behaviors led to their HIV status.^28^ Such statements may also be a manifestation of provider burnout and compassion fatigue, known to be global phenomena in the COVID-19 pandemic.^18^^–^^20^ Additional research is needed to explore how previously existing stigmas among service providers related to HIV status and substance use may synergistically interact with and be further reinforced by compassion fatigue and new COVID-19-related stigmas.

The COVID-19 pandemic remains a prominent threat in Kazakhstan, whose population was only 26% fully vaccinated as of August 2021.^29^ With service limitations likely to continue for some time, our findings hold immediate implications for NSPs and AIDS Centers. These organizations should maintain flexible solutions to ensure continuous service delivery, including delivery of ART to clients through NSPs. Continuing these services even beyond the pandemic may increase PWID adherence and retention in care. There is also an urgent need for NSPs and their staff to address overdose prevention and scale up MOUD services, and for Kazakhstan's Ministry of Health to collect and disseminate accurate and updated surveillance data on overdose among PWID. Furthermore, neither COVID-19 testing nor vaccination is currently provided through NSPs, as they have been in other countries.^21^ Kazakhstan's polyclinics require official identification documents for testing and vaccination,^30^^,^^31^ which may discourage PWID from seeking these services at these locations. Allowing NSPs (which serve clients without ID) to assume these responsibilities may remove some burden from traditional medical facilities like polyclinics and may increase the accessibility of these services to marginalized groups like PWID. Finally, providers should receive consistent adequate pay (rather than short-term bonuses) and psychological support to offset the financial and emotional burdens of working under pandemic conditions.

### Limitations

Although we randomly selected Bridge study participants to take part in these in-depth interviews, the parent study sample itself was not random. Bridge NSPs and staff were selected for inclusion based on criteria related to the parent study,^26^ which limits the representativeness of our sample and the generalizability of our results. All interviewees had overseen or implemented a case management intervention for PWID over the past 2–3 years and therefore may have been accustomed to finding creative solutions for PWID outreach and retention. Staff at NSPs and AIDS Centers who did not participate in the parent study may have had different attitudes and approaches to service provision during the pandemic. Therefore, our results may not be generalizable beyond the study sample.

Secondly, participation in interviews occurred from May to August 2020 while many changes in lockdown status occurred (Figure). Our interviews only captured participant perspectives at a single moment in time, though their experiences may have continued to evolve as the pandemic continued.

We did not interview outreach workers about their experiences providing field-based services during the pandemic. Therefore, our findings are limited to their supervisor's perspectives on their work and efforts. Finally, we did not conduct interviews with PWID clients of harm reduction programs to understand their experiences during the pandemic and how they navigated service changes. This particularly limits our findings regarding changes in drug use, HIV, and COVID-19-related risks among PWID. Without client perspectives on COVID-19, HIV, and substance misuse risks, it is difficult to ascertain the accuracy of provider perceptions regarding these risks.

## CONCLUSION

As the COVID-19 pandemic continues, the government of Kazakhstan must ensure that key populations such as PWID are not excluded from the response, which includes both HIV and COVID-19 prevention efforts as well as efforts to continue the delivery of essential services. Our findings demonstrate the challenges facing providers in harm reduction settings during this time, as well as the creative solutions that staff are employing in the face of low resources and a rapidly changing environment. By investing in these organizations, governments can support this work and protect a vulnerable population.

## Notes

**Peer Reviewed**

**First published online:** April 8, 2022.
